# Owner’s Perspective About the Use of Mirtazapine Transdermal Ointment in Cats—A Survey-Based Study

**DOI:** 10.3390/ani15203054

**Published:** 2025-10-21

**Authors:** Sofia Carvalho, Beatriz Mendoza, Isabella Tirelli, Andrea Corsini, Rodolfo Oliveira Leal

**Affiliations:** 1 Hospital Escolar Veterinário, Faculty of Veterinary Medicine, University of Lisbon, 1300-477 Lisbon, Portugal; sofiaisabelfc@gmail.com; 2 IVC Evidensia, Hospital Veterinário Bom Jesus, 4715-380 Braga, Portugal; bmendoza@fmv.ulisboa.pt; 3 CIISA—Centre for Interdisciplinary Research in Animal Health, Faculty of Veterinary Medicine, University of Lisbon, Clinics, 1300-477 Lisbon, Portugal; 4 Associate Laboratory for Animal and Veterinary Sciences (AL4AnimalS), 1300-477 Lisbon, Portugal; 5 Department of Veterinary Science, University of Parma, 43121 Parma, Italy; isabella.tirelli@unipr.it (I.T.); andrea.corsini@unipr.it (A.C.)

**Keywords:** appetite stimulant, survey, anorexia, chronic disease, perception

## Abstract

**Simple Summary:**

Mirtazapine is currently used in feline medicine to improve appetite in cats. In this study, we aimed to assess cat owner’s feedback about the use of transdermal mirtazapine. A multicentric survey-based study was conducted. A total of 70 owners answered a thorough survey about how easy it was to give, possible side effects, and their general perception about mirtazapine. Most owners reported that the application was easy, considering it effective in stimulating their cats’ appetite. Some side effects were noted, such as increased vocalization, redness of the ear, or restlessness, but these were not frequent. Few owners had used oral mirtazapine before, and most of them found the administration of transdermal formulation easier. However, some preferred the oral form due to its lower cost. The results of this study show that the use of transdermal mirtazapine is generally well accepted by owners, being an apparent compliant medication in feline medicine.

**Abstract:**

Mirtazapine is an antidepressant used as an appetite stimulant in cats. This study aims to assess owner perspectives on the use of transdermal mirtazapine in cats. A multicentric survey-based study was conducted. A survey of 15 questions about efficacy, side effects, and overall perception was sent to owners of cats that had received the treatment between January 2021 and March 2023 in two European veterinary hospitals. From 108 contacted owners, 70 responses were obtained. Application to the ear was considered easy by 97% of respondents, and 91% followed the manufacturers’ instructions for alternating ears. Side effects were reported by 20%, most often increased vocalization, redness, or restlessness. Chronic kidney disease was the most common reason for prescription. Nine owners had previously used the oral form, and most of them found the transdermal option easier, although some preferred the oral version due to lower cost. Overall, 77% of owners considered the treatment effective in stimulating appetite. Regarding length of therapy, about half administered it for less than 14 days, while the rest followed the labeled duration, with longer use associated with more consistent appetite improvement. These findings highlight that transdermal mirtazapine is generally well accepted by owners, easy to administer, and effective in promoting appetite in cats.

## 1. Introduction

Inappetence and anorexia are nonspecific clinical signs very common in feline veterinary medicine [1,2], frequently being the first clinical sign identified by caregivers [3,4].

Pharmacological appetite stimulation may be useful in some circumstances, but should never replace or delay the implementation of other forms of nutritional management (e.g., feeding tubes/early parenteral nutrition when indicated) [5]. Historically, various medications have been used for appetite stimulation, including cyproheptadine and B-complex vitamins, among others [4,5]. However, currently, only two medications are licensed for use in cats: oral capromorelin (Elura, Elanco, available in the USA) and transdermal mirtazapine (Mirataz, Dechra, available in the USA, Canada, and Europe) [4].

Mirtazapine, a tetracyclic antidepressant, blocks presynaptic alpha-2-adrenergic on both noradrenergic and serotonergic neurons, thus facilitating the release of norepinephrine (NE) and serotonin (5-HT) into the synapse [6,7,8]. Additionally, it directly blocks postsynaptic serotonergic receptors type two and three (5-HT2 and 5-HT3) [6,7,8].

Mirtazapine was licensed in 2019 to be used as a transdermal ointment in cats, notwithstanding previous off-label oral administration in this species [9]. Several studies have demonstrated mirtazapine’s efficacy in weight gain and increasing appetite in cats [10,11,12,13,14,15].

The side effects associated with transdermal administration are similar to those observed with oral mirtazapine. However, local reactions, such as erythema, dryness, or pruritus at the site of application, may occur [16]. Mirtazapine (Mirataz, Dechra) contains macrogol 3350 as an excipient [17]. This excipient, also known as polyethylene glycol (PEG) 3350, has been identified as a potential allergen [18]. The product information of mirtazapine (Mirataz, Dechra) [9] indicates local application reactions (erythema, scabs, dryness, peeling, and itching) and behavioral changes (increased vocalization, hyperactivity/agitation, disorientation, lethargy, and aggressiveness) occurred very frequently in both safety and clinical studies. Depending on the severity of vomiting, dehydration, or behavioral changes, the administration of transdermal mirtazapine may be interrupted according to the benefit-risk assessment conducted by the veterinarian.

In the mirtazapine (Mirataz, Dechra) Summary of Product Characteristics (SPC) [9], the labeled dose for cats is 2 mg of mirtazapine per cat (0.1 g of ointment per cat, equivalent to a 3.8 cm line), administered once a day for 14 consecutive days. The product should be applied to the inner surface of the ear, with the application alternating between the ears each day, for example, the right ear one day and the left ear the next day.

Topical products offer several advantages, including ease of application and reduced systemic side effects [19]. However, the success of these treatments heavily relies on owner compliance. To the best of the authors’ knowledge, there have been no studies investigating and analyzing the perspectives of owners who have administered transdermal mirtazapine to their cats. This study aims to bridge this critical gap by assisting veterinarians in better understanding owner experiences and optimizing treatment outcomes. Therefore, the primary objective of this research is to investigate and analyze the feedback from cat owners regarding the use of transdermal mirtazapine ointment, including the ease and correctness of application (daily application to alternating ears and adherence to the labeled treatment duration). The secondary objectives are to explore common prescription patterns, common adverse effects reported, whether owners prefer it over mirtazapine pills for human use, assess if they found transdermal mirtazapine effective in stimulating appetite, and evaluate the perceived time until efficacy. We also aimed to assess whether there is a possible relationship between owner perceived efficacy and treatment duration. We hypothesize that transdermal mirtazapine is perceived by owners as a practical and effective treatment option, associated with few adverse effects. This information will aid veterinarians in better understanding owner experiences and optimizing treatment outcomes.

## 2. Materials and Methods

A descriptive cross-sectional retrospective survey-based study was conducted online using the electronic platform Google Forms^®^. The questionnaire comprised 15 questions (13 multiple-choice and 2 open-ended) and is available as a Supplementary Material File S1. The survey development process involved careful consideration of the questions to ensure comprehensive coverage of various aspects of transdermal mirtazapine use, including its efficacy, adverse effects, and overall perception. After an initial screening conducted by authors, the survey was assessed by a small group of veterinarians to ensure clarity, language consistency and relevance of questions. The questionnaire was then translated into Portuguese and Italian by native-speaking authors. Small adjustments were made to improve linguistic and cultural appropriateness. The final survey was then distributed among cat owners whose cats were treated at two European Veterinary Teaching Hospitals (Hospital Escolar Veterinário from FMV-ULisboa and University Veterinary Teaching Hospital of Padova) and were prescribed transdermal mirtazapine during the period from March 2021 to January 2023. Owners were contacted Via email or telephone to invite them to participate in the study. All the responses were submitted exclusively online Via the Google Forms^®^ platform (post-2016 online version/Google Workspace).

Participation in the survey was voluntary, with no incentives offered. Prior to engaging in data collection, each respondent had the option to agree or disagree with the following statement: “My participation in this survey is free and voluntary. I authorize the data to be used exclusively for statistical purposes, and my identity will not be disclosed”.

Perceived efficacy was assessed as a subjective measure, based on owners’ feedback of any improvement in appetite, without standardized criteria or specific behavioral or feeding indicators to categorize their observations. The survey did not include questions about the transitory nature or intensity of adverse effects. Since the labeled treatment duration is 14 days, for statistical analysis, responses were categorized into two groups: cats that received transdermal mirtazapine for less than 14 days (‘<14 days’) and cats that received it for 14 or more days (‘≥14 days’). The ‘reasons for the prescription of mirtazapine’ were obtained using a comprehensive database with the patient’s medical history and an open-ended question where owners recalled the grounds for the prescription. For a better analysis, answers were categorized by organ/disease (e.g., ‘liver disease’ encompasses hepatobiliary diseases such as triaditis, cholangitis, cholecystitis, acute hepatitis, and chronic hepatitis).

All data were collected using Google Forms^®^ and imported into a Microsoft Excel^®^ 2016 database. Descriptive statistics were generated in Microsoft Excel^®^ 2016 to summarize the data. Statistical analysis was conducted using IBM SPSS^®^ Statistics for Windows, version 28.0.1.0, with a significance level set at *p* < 0.05 for a 95% confidence interval in all tests. When appropriate, *χ^2^* and Fisher’s Exact tests were used to evaluate owners’ responses and explore potential associations between variables. The analysis was exploratory in nature, and examined relationships included perceived efficacy versus treatment duration, perceived efficacy versus underlying disease for prescription, and adverse effects versus ear alternation. Data were mainly descriptive, and no multiple comparisons were performed. Missing data were excluded on a per-question basis.

## 3. Results

### 3.1. Survey Response

Out of a total of 108 contacted owners (68 from Portugal and 40 from Italy), 71 responses were received (33 from Portugal and 38 from Italy), resulting in an overall response rate of 65.7% (71/108).

The first question of the questionnaire addressed the owner’s recall of using transdermal mirtazapine. One respondent indicated not remembering using the ointment; therefore, for statistical analysis, only the 70 responses from owners who confirmed mirtazapine (Mirataz, Dechra Pharmaceuticals PLC, Northwich, UK) use were included.

### 3.2. Perceived Efficacy

**Figure 1:**
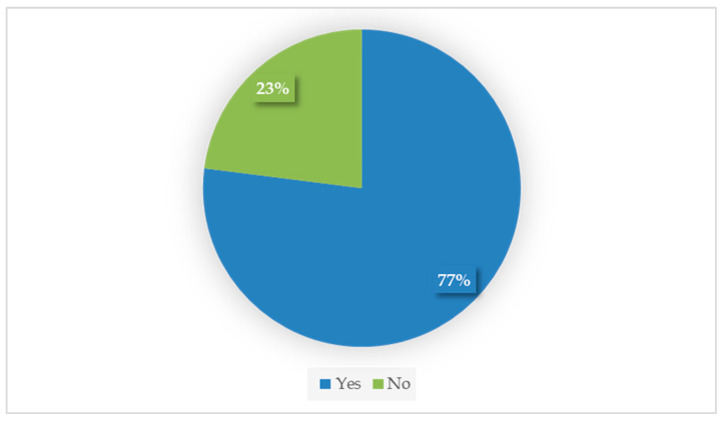
Owner’s main perception about transdermal mirtazapine’s efficacy (yes vs. no; n = 70).

A total of 77% (54/70) of respondents considered that transdermal mirtazapine efficiently stimulated the appetite of their cats (Figure 1). Owners reported observing positive effects at different time points: 40.7% (22/54) noticed an increase in appetite within 24 h, 50% (27/54) within less than one week, 11.1% (6/54) after one week, and 3.7% (2/54) could not recall the exact time until appetite returned.

### 3.3. Application

The topical application on the inner ear was considered easy by 97.1% (68/70) of respondents. The two owners who found the application difficult attributed it to their cat’s behavior (2/2) and ear debris (1/2). Adherence to the manufacturer’s instructions was high, with 91.4% (64/70) applying the ointment in alternating ears, whereas 8.6% (6/70) applied it in the same ear each day.

### 3.4. Adverse Effects

Adverse effects were reported by 20% (14/70) of owners. The most common effects included:

- Agitation: 8.6% (6/70)
- Erythema: 7.14% (5/70)
- Vocalization: 5.7% (4/70)

**Figure 2:**
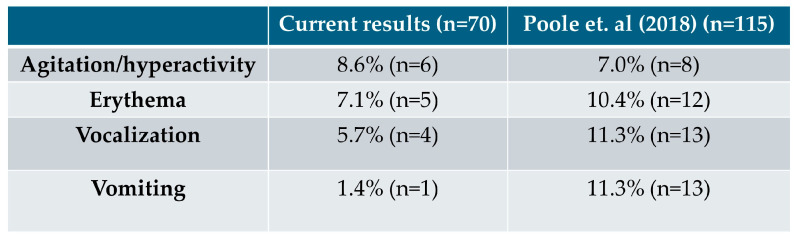
Comparison of side effects reported in our study and in a study by Poole Et Al. (2018) [15].

Figure 2 illustrates the disparities in the reported percentages of side effects between our study and the data from a study by Poole Et Al. (2018) [15], included on the mirtazapine (Mirataz, Dechra) Summary of Product Characteristics (SPC) (2020) [9].

### 3.5. Underlying Disease for Prescription

Among 57 responses detailing underlying diseases (81% of 70), the most frequent causes of anorexia were:

- Chronic kidney disease: 32% (18/57)
- Chronic enteropathy: 18% (10/57)
- Liver disease: 12% (7/57)
- Post-surgical recovery: 9% (5/57)
- Neoplasia: 7% (4/57)

**Figure 3:**
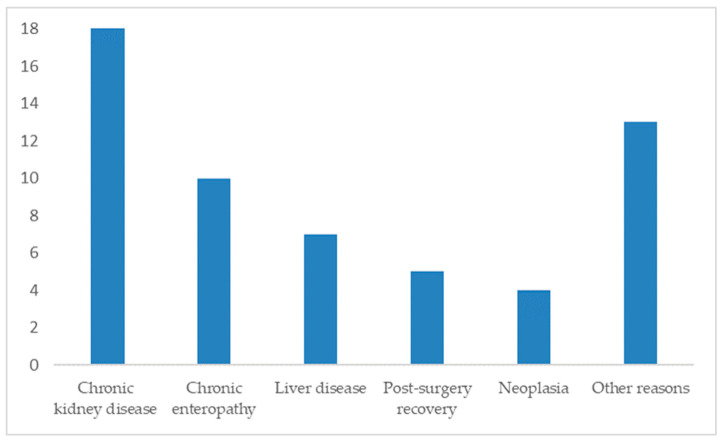
Distribution of cats by reason of prescription of transdermal mirtazapine ointment (n = 57).

Other causes included chronic pancreatitis, gastroenteritis, respiratory disease, cystitis, FeLV, idiopathic hypercalcemia, intestinal malformation, leukocytosis, and lymphadenopathy (Figure 3).

### 3.6. Previous Use of Oral Mirtazapine

Nine owners (13%) had previously administered oral mirtazapine. Of these, 78% (7/9) considered transdermal more beneficial due to easier administration, while 22% (2/9) preferred oral mirtazapine due to lower cost.

### 3.7. Statistical Analysis of Variable Relationships

In the statistical analysis of variable relationships, we examined cat owners’ responses to identify potential correlations. The explored relationships included perceived efficacy versus treatment duration, perceived efficacy versus underlying disease for prescription, and adverse effects versus ear alternation. No statistically significant associations were found for perceived efficacy versus underlying disease or for adverse effects versus ear alternation (for example, there was no significant association between not alternating ears and erythema); the only significant relationship observed was between treatment duration and perceived efficacy.

#### Treatment Duration and Efficacy

**Figure 4:**
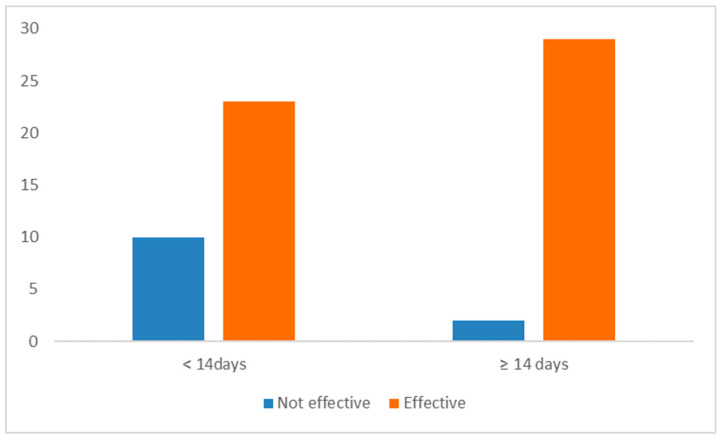
Perceived efficacy of transdermal mirtazapine according to treatment duration (short-term/less than 14 days versus long-term/14 days or longer) (n = 64).

Responses on treatment duration were categorized into two groups: ‘<14 days’ (51.6%, 33/64) and ‘≥14 days’ (48.4%, 31/64). A statistically significant association (*p* = 0.015) was observed between treatment duration and perceived efficacy. Owners administering treatment for ≥14 days reported a higher positive outcome (93.5%) compared to those administering it for <14 days (69.7%) (Figure 4).

## 4. Discussion

This study provided new insights into owners’ perception of transdermal mirtazapine in cats.

Our study found that owners generally consider the ointment easy to apply, highlighting its advantages over traditional pills. Administering oral medications to cats can be challenging due to their behavior and susceptibility to stress [20]. Therefore, the simplicity of ointment application not only improves compliance [21], but also reduces feline stress levels during treatment. This emphasizes the importance of versatile and stress- minimizing treatment options for the well-being of feline patients. Incorporating a question about whether owners experienced challenges in accurately measuring the prescribed dose (using the 3.8 cm line) would have been an interesting addition to the survey. The correct application of topical treatments is paramount to their efficacy, especially in cases where owners are responsible for administering the product at home. Enhancing communication between veterinarians and cat owners is crucial for ensuring the accurate administration of treatments. Although most owners do appropriately rotate administration between both ears, a minor percentage still apply the ointment to the same ear consistently, indicating room for improvement in daily practice communication. While concerns such as increased irritation or buildup of material potentially affecting absorption could arise from repeated application to the same ear, no statistically significant association was found between ear irritation and the exclusive use of one ear for application.

Regarding adverse effects, specifically erythema, it is important to note that mirtazapine (Mirataz, Dechra) contains macrogol 3350 as an excipient SPC) [9]. The presence of PEG 3350 in this ointment may contribute to the occurrence of erythema, as these compounds have been associated with allergic reactions, including skin irritation and erythema in some individuals [18]. Regarding vomiting, it would have been valuable to better differentiate between true vomiting and regurgitation, as increased appetite stimulation may lead some cats to eat too quickly and subsequently regurgitate rather than vomit. Additionally, differentiating vomiting associated with the underlying condition from vomiting potentially related to mirtazapine (Mirataz, Dechra) administration would have provided a clearer understanding of the drug’s adverse effects. Side effects were reported by 20% cat owners (14/70), with agitation/hyperactivity being the most frequent. Compared to Poole et al. (2018) [15], where agitation/hyperactivity occurred in 7%, erythema in 10.4%, and vocalization and vomiting in 11.3%, our study showed a slightly higher percentage of agitation, lower percentages of erythema and vocalization, and a markedly lower incidence of vomiting (1.4% vs. 11.3%). This comparison highlighted differences between our findings and previous clinical studies, providing insight into the real-world use of transdermal mirtazapine in cats.

Mirtazapine is recognized as a medication designed to promote an increase in body weight in cats experiencing long-term conditions associated with poor appetite and weight loss. The responses from our survey highlighted chronic kidney disease as the predominant reason for prescribing transdermal mirtazapine, followed by chronic enteropathy and liver disease. Furthermore, our results highlight the versatility of transdermal mirtazapine, extending its application to more acute scenarios like post-surgical recovery.

The comparison of owners who had previous experience using both formulations of mirtazapine (ointment and pills) (9/70) revealed interesting insights into their preferences. Although this subset was relatively small (9/70; 13%), a substantial majority (7/9; 78%) expressed a preference for the transdermal ointment in contrast to a minority of respondents (2/9, 22%) who preferred oral mirtazapine. These preferences highlight the individualized nature of cat responses and owner perceptions, reflecting the multifaceted considerations that influence the choice between oral and transdermal mirtazapine administration.

Most owners reported an increase in their cat’s appetite in less than a week, with around 40% of the cases showing a positive response in the first 24 h. Nonetheless, the variations in perceived time until efficacy (improvement in appetite) highlight the individualized nature of feline responses to transdermal mirtazapine and the variability in owners’ perceptions. It is also important to note that many cats likely received additional therapies for their underlying diseases, which could have influenced the time to efficacy. Furthermore, cats with different underlying conditions and varying disease severities were included, adding to this variability. While pharmacokinetic parameters such as Tmax provide valuable data on drug concentration dynamics, the time until perceived efficacy reported by owners reflects the broader clinical impact, incorporating both the physiological response to appetite stimulants and the subjective nature of owner interpretations.

The observed distribution of treatment durations provided insights into the real-world use of transdermal mirtazapine. In contrast to the labeled 14-day duration in the official product information, our study reveals variations in how long cat owners administered the ointment. Approximately 51.6% of owners used it for fewer than 14 days, indicating deviations from the labeled duration. The study also suggested higher perceived efficacy in cats treated for more than 14 days and lower perceived efficacy in those treated for fewer than 14 days, implying that treatment duration may influence perceived efficacy. Another possible explanation is that owners who found the treatment effective were more likely to continue using it, whereas those who perceived it as ineffective may have discontinued use earlier, leading to shorter treatment durations. Additionally, treatment duration may be influenced by factors such as the underlying disease, severity of anorexia or weight loss, veterinarian recommendations, and owner perceptions of improvement or adverse effects. Nonetheless, this statistical association (*p* = 0.015) should be interpreted cautiously, considering the small sample size and the absence of adjustment for potential confounders. Further research with larger sample sizes and standardized treatment protocols could provide a more comprehensive understanding of this relationship.

Although this study provides valuable insights into cat owners’ perspectives on the use of transdermal mirtazapine, it is essential to acknowledge certain limitations. The analysis was based on a relatively small sample of 70 responses, which may have restricted the extent of conclusions that can be drawn from the data. The study was conducted in specific geographical areas (Portugal and Italy) so these findings might not apply universally to all cat owners globally.

While most owners reported satisfaction with transdermal mirtazapine appetite-stimulating effects, efficacy was assessed using a yes/no question rather than a standardized scale, making it inherently subjective. Owner perceptions may have been influenced by expectations, observation bias, or placebo effects, and thus differ from actual clinical efficacy. Measuring objective outcomes, such as changes in body weight during treatment, could have provided a more accurate assessment of clinical effectiveness in future studies.

Another limitation of this study was that adverse effects were not evaluated using a standardized scale for intensity or duration. Owners were only asked to report whether any adverse effects occurred, without specifying their severity or persistence. Furthermore, the survey did not include a question about whether these effects were resolved after treatment discontinuation.

It is worth noting that some owners who participated in the survey had used the transdermal ointment as far back as 2021 and were responding to the survey in 2023. This significant gap between the time of application and the survey response may have introduced the possibility of a recall bias, in which owners may struggle to accurately remember details about their experiences with the medication. Such gaps in memory can lead to variability and inaccuracies of their responses, potentially affecting the reliability of the data collected. To mitigate this limitation, future research could benefit from a prospective study design, which involves collecting data in real-time or shortly after the medication’s use. Furthermore, there is a possibility for variations in the interpretation of survey questions. The clarity and wording of these questions could influence the accuracy of responses. Additionally, there is potential for response bias, as owners who experienced significant effects, whether positive or negative, with mirtazapine may have been more motivated to participate, thereby introducing selection bias.

Another limitation of our study pertains to the question regarding the time until efficacy of transdermal mirtazapine. Presenting this question in a free-response format may have inadvertently resulted in a wide variety of responses. In retrospect, providing predefined options could have facilitated subsequent statistical analysis.

The lack of veterinary involvement in this study was a significant limitation. Veterinary input could have been beneficial in several areas, particularly in assessing adverse effects and efficacy. One notable limitation of the study relates to the assessment of adverse effects, as these were solely evaluated based on the owners’ recollection. To enhance the objectivity and precision of side effect reporting, future research could benefit from incorporating a standardized scale.

Greater access to medical records would have improved data accuracy, particularly regarding underlying diseases, anorexia causes, and mirtazapine prescriptions. Relying on owners’ recollections may have introduced recall bias, affecting the reliability of findings. Medical records would have allowed for better categorization of conditions and a clearer understanding of factors influencing treatment outcomes. The study also did not explore variations in medication dosages, prescribed regimens, other medications, or the severity of the cats’ conditions, all of which could influence treatment efficacy.

Furthermore, the survey could have been enhanced by including questions related to owners’ self-assessment of their compliance with treatment regimens and the reasons for any reported non-compliance. Additionally, the study did not incorporate a scale for cat owners to indicate their overall satisfaction levels with the use of transdermal mirtazapine. These additions could have provided a more comprehensive understanding of owner experiences and satisfaction with this treatment.

## 5. Conclusions

This study provides insights into cat owners’ perceptions of transdermal mirtazapine, indicating that it is generally well accepted and perceived as effective in stimulating appetite. Adverse effects were infrequently reported and mostly mild. A significant association between treatment duration and perceived efficacy was observed, highlighting an area for further investigation. These findings contribute to understanding real-world use and suggest directions for future research, including objective measures of clinical outcomes.

## Data Availability

The original survey in this study is included in the Supplementary Material. Further inquiries can be directed to the corresponding author.

## Supplementary Materials

The following supporting information can be downloaded at: https://www.mdpi.com/article/10.3390/ani15203054/s1. The owner’s perspective questionnaire used in this study is available in Supplementary File S1.

## Abbreviations

The following abbreviations are used in this manuscript:

5-HT	Serotonin or 5-hydroxytryptamine
CBC	Complete Blood Cell Count
CKD	Chronic Kidney Disease
Cmax	Maximum Concentration
CTM	Compounded Transdermal Mirtazapine
FeLV	Feline Leukemia Virus
PEG	Polyethylene Glycol
Tmax	The time it takes for a drug to reach the maximum concentration (Cmax)
