# Supplementary material for: Owner’s Perspective About the Use of Mirtazapine Transdermal Ointment in Cats—A Survey-Based Study
Source: Animals (Basel). 2025 Oct 21;15(20):3054. doi: 10.3390/ani15203054 (PMC12562069; doi:10.3390/ani15203054)
Supplement: Supplementary file 1 [file animals-15-03054-s001.zip › animals-3919165-supplementary File S1.pdf]

animals

MDPI

Supplementary Material

1 Supplementary Data

The survey - **Use of Mirataz - owners' perspective**

Mirataz is an ointment, applied to the ear, which aims to stimulate the appetite of cats.

Your response will contribute to the advancement of science allowing us to evaluate owners' perception of Mirataz and optimise this treatment in future cases.

This questionnaire takes about 2 minutes to answer. We thank you very much for your participation.

Responsible for the study: Sofia Carvalho (student of the 6th year of the Integrated Master in Veterinary Medicine at University of Lisbon)

Supervisor of the study: Professor Rodolfo Oliveira Leal

If you have any questions or want some clarification, you can contact me via my email: sofiaicarvalho@campus.ul.pt

1. My participation is free and voluntary. I agree that the data will be used exclusively for statistical purposes and my identity will not be disclosed.

2. Email?

3. Do you remember using Mirataz?

   a. Yes

   b. No

4. Was the application of the ointment easy?

   a. Yes

   b. No

5. What was the reason? (if you answered no to the previous question)

  a. The ear was too dirty
  b. It was difficult to dose the ointment
  c. My cats behaviour didn’t allow me to use the ointment
  d. Other:________

6. Did you change the ear in which you put the ointment? (One day on his left ear, the next day on his right ear, and so on)

  a. Yes
  b. No, I applied it always on the same ear

7. How many days did you apply the ointment?

  a. 1 week
  b. 2 weeks
  c. 3 weeks
  d. 1 month
  e. More than 1 month
  f. My cat is using Mirataz continuously
  g. I don’t remember
  h. Other_______

8. Does your pet still use Mirataz at the moment?

  a. Yes
  b. No

9. Have you noticed any side effects associated with Mirataz?

a. Yes

b. No

10. If yes, which ones?

a. Vocalization

b. Agitation

c. Vomiting

d. Diarrhea

e. Redness on the ear

f. Excessive urination

g. Hypersalivation/ tremors

h. Other________

11. Do you think the ointment worked, stimulating the appetite?

a. Yes

b. No

12. How long after the start of the applications did you notice improvements in appetite?

____

13. Has your cat ever taken mirtazapine orally (in the form of a pill)?

a. Yes

b. No

14. Do you think that the ointment is better than pills?

a. Yes

b. No

15. In your view, what are the main advantages of ointment over pills?

    a. I don’t like giving pills to my cat
    b. The ointment is easy to apply
    c. I think that my cat restored the appetite faster with the ointment
    d. Other__________

16. In your view, what are the main disadvantages of ointment over pills?

    a. The ointment is more expensive
    b. I think that my cat restored the appetite faster with pills
    c. I found it difficult to apply the ointment on the ears
    d. Other_________

17. Thank you for your participation!
